# An immune risk score predicts progression-free survival of melanoma patients in South China receiving anti-PD-1 inhibitor therapy—a retrospective cohort study examining 66 circulating immune cell subsets

**DOI:** 10.3389/fimmu.2022.1012673

**Published:** 2022-12-07

**Authors:** Peidong Chi, Hang Jiang, Dandan Li, Jingjing Li, Xizhi Wen, Qiyue Ding, Linbin Chen, Xiaoshi Zhang, Junqi Huang, Ya Ding

**Affiliations:** ^1^ Sun Yat-sen University Cancer Center, State Key Laboratory of Oncology in South China, Collaborative Innovation Center for Cancer Medicine, Guangzhou, China; ^2^ Department of Clinical Laboratory, Sun Yat-Sen University Cancer Center, Guangzhou, Guangdong, China; ^3^ Department of Biotherapy Center, Sun Yat-Sen University Cancer Center, Guangzhou, Guangdong, China; ^4^ Organ Transplant Center, The First Affiliated Hospital, Sun Yat-sen University, Guangzhou, China; ^5^ Guangdong Provincial Key Laboratory of Organ Donation and Transplant Immunology, Guangzhou, China; ^6^ Guangdong Provincial International Cooperation Base of Science and Technology (Organ Transplantation), Guangzhou, China; ^7^ Department of Laboratory Medicine, The First Affiliated Hospital, Sun Yat-sen University, Guangzhou, China

**Keywords:** melanoma, PD-1, immune checkpoint blockade inhibitor therapy, innate immune, adaptive immune

## Abstract

**Introduction:**

Immune checkpoint blockade inhibitor (ICI) therapy offers significant survival benefits for malignant melanoma. However, some patients were observed to be in disease progression after the first few treatment cycles. As such, it is urgent to find convenient and accessible indicators that assess whether patients can benefit from ICI therapy.

**Methods:**

In the training cohort, flow cytometry was used to determine the absolute values of 66 immune cell subsets in the peripheral blood of melanoma patients (n=29) before treatment with anti-PD-1 inhibitors. The least absolute shrinkage and selection operator (LASSO) Cox regression model was followed for the efficacy of each subset in predicting progression-free survival. Then we validated the performance of the selected model in validation cohorts (n=20), and developed a nomogram for clinical use.

**Results:**

A prognostic immune risk score composed of CD1c^+^ dendritic cells and three subsets of T cells (CD8^+^CD28^+^, CD3^+^TCRab^+^HLA-DR^+^, CD3^+^TCRgd^+^HLA-DR^+^) with a higher prognostic power than individual features (AUC = 0.825). Using this model, patients in the training cohort were divided into high- and low-risk groups with significant differences in mean progression-free survival (3.6 vs. 12.3 months), including disease control rate (41.2% vs. 91.7%), and objective response rate (17.6% vs. 41.6%). Integrating four-immune cell-subset based classifiers and three clinicopathologic risk factors can help to predict which patients might benefit from anti-PD-1 antibody inhibitors and remind potential non-responders to pursue effective treatment options in a timely way.

**Conclusions:**

The prognostic immune risk score including the innate immune and adaptive immune cell populations could provide an accurate prediction efficacy in malignant melanoma patients with ICI therapy.

## Introduction

Malignant melanoma is one of the most aggressive diseases with a dismal prognosis. Data show that the incidence of malignant melanoma in China had a 110% rise compared with that in 1990 (1). Despite being at the forefront of personalized medicine, advanced/metastatic melanoma has very poor survival rates with traditional chemotherapy or cytokine therapy. In the past 10 years, with the continued exploration of targeted drugs and immunotherapy, e.g., BRAF inhibitors, MEK inhibitors, anti-CTLA-4, or anti-PD-1 antibodies, randomized studies showed that anti-PD-1 mAb treatment has shown improvement in the overall survival of metastatic melanoma patients by approximately fourfold, compared with dacarbazine treatment (2).

Although immunotherapies such as PD-1/PD-L1 could sometimes mediate complete, long-lasting responses, there are still a large number of patients who cannot achieve the expected therapeutic effect due to the low response rate (3). LDH status, tumor burden, mutation status, stage, and extent of primary disease were confirmed to be the most important prognostic indicators of malignant melanoma (4, 5). These clinicopathologic risk factors cannot differentiate patients who benefit from anti-PD-1 antibody therapy—so it appears that validated biomarkers as the new prognostic and predictive factors for the current staging system are necessary. Several potential predictors of anti-PD-1 antibody response with favorable outcomes were investigated, such as CTLA4 promoter hypomethylation, PD-L1 expression, mismatch repair deficiency, tumor mutation burden, tumor-infiltrating immune cell features, and circulating immune cells (6–9). Yet, their role in predicting treatment outcomes with anti PD-1 antibody is controversial and still requires validation.

Compared with detecting biomarkers from tumor tissue, peripheral blood offers better accessibility. Several studies analyzed the clinical relevance and prognostic value of circulating lymphocyte subsets in malignant tumors (9–12), whereas some only indicated the importance of immune cells and did not specify precise subsets. Compared with a single biomarker, integrating multiple biomarkers into a single model would better shape immune phenotypes. Although multicolor flow cytometry, coupled with high-dimensional analysis, offers an opportunity to study circulating immune cell subsets in detail (13), the analysis was not without challenges. When the number of covariates is close to or greater than the number of observations, the Cox proportional hazard regression analysis, the most popular approach to model covariate information for survival times, is no longer suitable (14). Moreover, 66 variables and 49 specimens were recruited in this study, so conventional bivariate analysis would dilute its statistical power to detect differences. We developed the Least Absolute Shrinkage and Selection Operator (LASSO) method to eliminate this issue (15, 16). The LASSO Cox regression model combined with a four-immune cell-subset-based classifier could predict progression-free survival and enable patients with stage III/IV malignant melanoma to benefit from the anti-PD-1 antibody.

## Patients and methods

### Patients

This study follows a single-center retrospective cohort design at Sun Yat-sen University Cancer Center (SYSUCC). We addressed these issues by analyzing two retrospective cohorts (training and validation cohorts) of patients with stage IIIc/d or IV melanoma (AJCC 8th) with an indication toward anti-PD-1 treatment from May 2020 to March 2021. All patients had pathologic confirmation of melanoma diagnosis by an experienced pathologist and agreed that their data were to be used for research; it was conducted in accordance with the principles of the Helsinki Declaration. The Ethics Committee of SYSUCC reviewed and approved the study (Reference No. SZR2019-097). All participants obtained informed consent, but we excluded patients receiving only one course of anti-PD-1 treatment or not having a complete workup after treatment. All patients had an Eastern Cooperative Oncology Group (ECOG) score of 0 or 1.

### Flow cytometry detection for immune cell subsets

In the training set, we adopted the DURAClone IM immune function reagent (Beckman Headquarters, Brea, CA, USA) that included 50 antibodies distributed in six tubes to identify immune cell subsets with flow cytometry (**Table S1**). Peripheral blood of melanoma patients was collected with heparin sodium as an anticoagulant before and after receiving the anti-PD-1 inhibitor. Fluorochrome-conjugated anti-human monoclonal antibodies were DURAClone dry reagents, obtained from Beckman Coulter (Marseille, France), except anti-CD127, the liquid reagent. Six panel matrices were defined for 8- to 10-fluorochrome channels. Cell staining was performed within 4 h after blood collection, whereas 100 μl of anticoagulated peripheral blood was stained with surface antibodies for 15 min at room temperature in the dark prior to lysis with OptiLyse C No-Wash Lysing Solution (Beckman Coulter). Lysed cells were washed twice with PBS prior to acquisition. For the detection of B-cell subsets, 100 μl of anticoagulated peripheral blood was washed twice with PBS before staining with B-cell markers for 15 min at room temperature in the dark with OptiLyse C No-Wash Lysing Solution (Beckman Coulter) and washed once with PBS prior to acquisition. All samples were measured with 10-color, three-laser Navios flow cytometers, as data files were analyzed with Kaluza software v. 1.2 (Beckman Coulter). A total of 66 circulating immune cell subsets were detected in this study, and detailed names of each cell population are shown in **Table S2**.

In the validation set, peripheral blood mononuclear cells (PBMCs) of melanoma were isolated by density gradient centrifugation, frozen in cryovials at a density of 5–10 × 10^6^ cells in 1 ml of freezing medium [(10% dimethyl sulfoxide (DMSO; Sigma-Aldrich, Burlington, MA, USA), 90% fetal bovine serum (FBS; Gibco/Fisher Scientific, Waltham, MA, USA)], and stored in liquid nitrogen. The staining protocol was used for the training set. Antibodies were anti-CD3-APC (clone SK7, BDIS, San Jose, CA, USA), anti-CD28-PE (clone CD28.2, BD Pharmingen, San Diego, CA, USA), anti-CD8-FITC (clone B9.11, Beckman), anti-TCRαβ-FITC (clone WT31, BDIS), anti-TCRγδ-PE (clone 11F2, BDIS), anti-HLA-DR-APC-Cy7 (clone L243, BDIS), anti-CD1c-PerCP-Cy5.5 (clone L161, BioLegend, San Diego, CA, USA), anti-CD11c-PE (clone 3.9, BioLegend), anti-CD123-PE-Cy7 (clone), and lineage cocktail reagents of anti-CD3-APC (clone SK7, BDIS), anti-CD19-APC (clone SJ25C1, BDIS), anti-CD20-APC (clone L27, BDIS), anti-CD14-APC (clone M5E2, BioLegend), and anti-CD56-APC (clone NCAM16.2, BDIS).

### Endpoint

We defined progression-free survival (PFS) as the time from the first anti-PD-1 treatment to confirmed tumor progression. Disease progression was assessed per RECIST 1.1. Imaging was performed before and after three treatment cycles or as deemed necessary.

### Statistics

The LASSO Cox regression model was adopted to achieve shrinkage and variable selection simultaneously (17). Ten-time cross validations were used to determine optimal values of λ. We calculated the risk score for each patient based on immune cell subsets, selected from LASSO Cox regression models, where risk score=coefficient X1*absolute count X1+coefficient X2*absolute count X2+…+coefficient Xn*absolute count Xn. For survival analyses, we used Kaplan–Meier to analyze the correlation between variables and progress-free survival and the log-rank test to compare survival curves. We investigated the prognostic accuracy of each feature and immune cell subset-based classifiers by using time-dependent receiver operating characteristic (ROC) analysis. The Cox regression model clarified the multivariable survival analysis. All statistical tests were done with R software (Core Team v. 4.0.5., Vienna, Austria), with statistical significance set at 0.05. Based on the Cox proportional hazard regression model, a prognostic nomogram was also performed to visualize the link between individual predictors and survival rates with the “rms” package. The C-index and calibration curves were used to evaluated the performance of the prognostic nomogram.

### Gene signature score validation

A single-cell RNA sequencing dataset (GSE120575) (18) was downloaded from the Gene Expression Omnibus (GEO) database (https://www.ncbi.nlm.nih.gov/geo/). The marker genes of the CD8^+^ T-cell, αβT-cell, γδT-cell, and DC clusters were identified by using “FindAllmarkers” functions (**Table S4**). The genes with adjusted P values <0.05 and logFC >1 were considered as significant and used for further analysis. The GSE120575 dataset contained 16,291 immune cells from 48 tumor samples of melanoma patients treated with checkpoint inhibitors. Downstream analyses including principal component analysis (PCA) and t-distributed stochastic neighbor embedding (UMAP) analysis were performed using the Seurat R package (19–22). The association between flow cytometry population ratios and feature gene signatures was utilized to validate the feature gene signatures under the presumption that the flow cytometry population ratios accurately reflect the genuine feature abundance in the patient sample. Four-immune cell-subset gene signature scores among cell clusters were calculated using the IOBR R package (23).

## Result

### Anti-PD-1 inhibitor outcome

Detailed clinicopathologic patient characteristics in the training (n = 29) and validation (n = 20) cohorts are shown in **Table 1**. The study population consisted of 32 women (65.3%, 32/49) and 17 men (34.7%, 17/49), median age 56 years (interquartile range, IQR 42–63). Most patients had the Eastern Cooperative Oncology Group (ECOG) score of 0 (65.3%) and a normal lactate dehydrogenase value (85.7%). In the total cohort, the most common primary tumor type was acral melanoma (44.9%, 22/49) followed by chronic sun damage (CSD, 36.7%, 18/49) and various other types (18.4%, 4/49), including one uveal melanoma, five mucosal melanomas, one anorectal melanoma, and two of unknown primary origins with metastasis. Ten patients (20.4%, 10/49) had unresectable stage III melanoma, and 39 (79.6%, 39/49) had stage IV. Sixteen patients (32.7%, 16/49) harbored a mutation in the BRAF V600E/K gene, and in five patients (10.2%, 5/49) it was found in the NRAS gene.

**Table 1:** Baseline characteristics of the training set and validation set.

Characteristic	Training set	Validation set
Age, median (IQR)^‡^	50 (39, 60)	59 (51,66)
Gender, n (%)		
Male	7 (24.1%)	10 (50.0%)
Female	22 (75.9%)	10 (50.0%)
ECOG, n (%)		
0	19 (65.5%)	13 (65.0%)
1	10 (34.5%)	7 (35.0%)
LDH, n (%)		
>ULN^*^	5 (17.2%)	2 (10.0%)
≤ULN	24 (82.8%)	18 (90.0%)
Histology, n (%)		
Acral	10 (34.5%)	12 (60.0%)
CSD^§^	15 (51.7%)	3 (15.0%)
Other	4 (13.8%)	5 (25.0%)
Stage, n (%)		
III	6 (20.7%)	4 (20.0%)
IV	23 (79.3%)	16 (80.0%)
BRAF V600E/K mutation status, n (%)		
Wild-type	14 (48.3%)	18 (90.0%)
Mutant	14 (48.3%)	2 (10.0%)
Unknown	1 (3.4%)	
NRAS mutation status, n (%)		
Wild-type	22 (75.9%)	17 (85.0%)
Mutant	2 (6.9%)	3 (15.0%)
Unknown	5 (17.2%)	
Regimen, n (%)		
PD-1 antibodies combined with chemotherapy	12 (41.4%)	9 (45.0%)
PD-1 antibodies combined with targeted therapy	12 (41.4)	7 (35.0%)
Other	5 (17.2%)	4 (20.0%)
Line of therapy, n (%)		
1L	21 (72.4%)	17 (85.0%)
≥2L	8 (27.6%)	3 (15.0%)
Immune risk score, n^††^ (%)		
High risk	15 (51.7%)	9 (45.0%)
Low risk	14 (48.3%)	11 (55.0%)

^‡^IQR, interquartile range. ^§^CSD, chronic sun damage. ^*^ULN, upper limit of normal.

^††^Immune risk score refers to the four-immune cell-subset-based classifier, including three subpopulations of T cells (CD8^+^CD28^+^, CD3^+^TCRαβ^+^HLA-DR^+^, CD3^+^TCRγδ^+^HLA-DR^+^) and CD1c^+^ dendritic cells.

Of 49 patients, six accepted anti-PD-1 antibody alone as first-line therapy, 40 (81.6%) received a combination of anti-PD-1 antibody treatment and chemotherapy or BRAF/BRAF-MEK inhibitor treatment, and three patients received a combination of anti-PD-1 antibody with anti-angiogenic agents. At the data cutoff, with a median follow-up of 12.5 months (range 2–17 months), patients received a median of 12 treatment cycles of anti-PD-1 antibody (range 3–23 cycles), for an objective response rate (ORR) of 26.4% (13/49). The median PFS for the entire cohort was 6.5 months (95% CI 4.9–8.1 months). One patient (2%) achieved a complete response. Among 49 patients, one patient (2%) achieved a complete response, 12 patients (24.4%) achieved a partial response, and 29 (59.1%) were stable, resulting in a disease control rate (DCR) of 85.8%. Thirty-one (63.2%) of 49 patients were in disease progression during the follow-up period.

### Construction of a prognostic model index (PI, risk score) based on immune cell subsets

**Figure 1:**
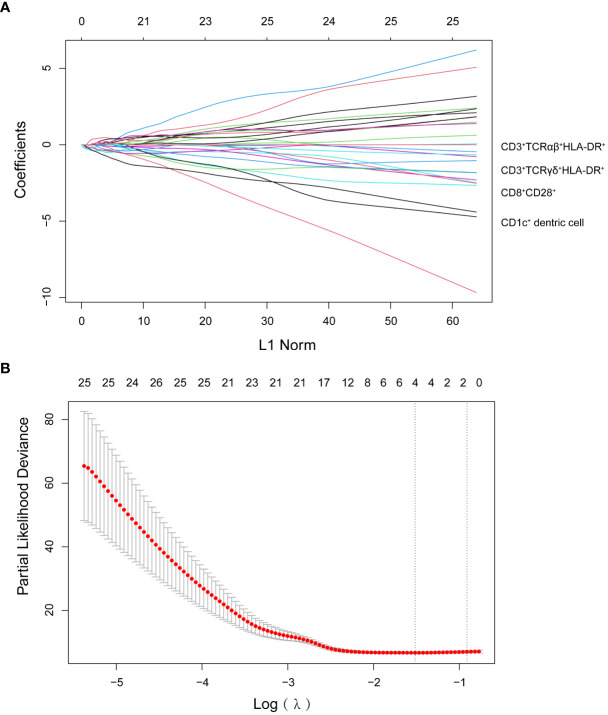
**(A)** LASSO coefficient profiles of the 66 lymphocyte subsets. **(B)** Ten-time cross-validation for tuning parameter selection in the LASSO model.

We detected 66 immune cell subsets in circulating blood specimens for 29 patients with malignant melanoma in the training set prior to therapy. We used a LASSO Cox regression model to build a prognostic classifier. We chose λ *via* minimal criteria and values λ = 0.22 and log λ = -1.51. We picked four different subgroups from the 66 immune cell subsets: CD1c^+^ dendritic cells (DC) and three subsets of T cells (CD8^+^CD28^+^, CD3^+^TCRαβ^+^HLA-DR^+^, CD3^+^TCRγδ^+^HLA-DR^+^) (**Figure 1**). As per LASSO Cox regression, we used the coxph (proportional hazards model) of the “survival” package to calculate a coefficient for every immune cell subset (24). We developed a formula for the risk score for each patient’s disease progression, with four-immune cell-subset absolute counts. Risk score = - (1.798*absolute count of CD8^+^CD28^+^ T cells) - (4.06*absolute count of CD3^+^TCRαβ^+^HLA-DR^+^ T cells) - (26.46*absolute count of CD3^+^TCRγδ^+^HLA-DR^+^ T cells) - (169*absolute count of CD1c^+^ dendritic cells).

**Figure 2:**
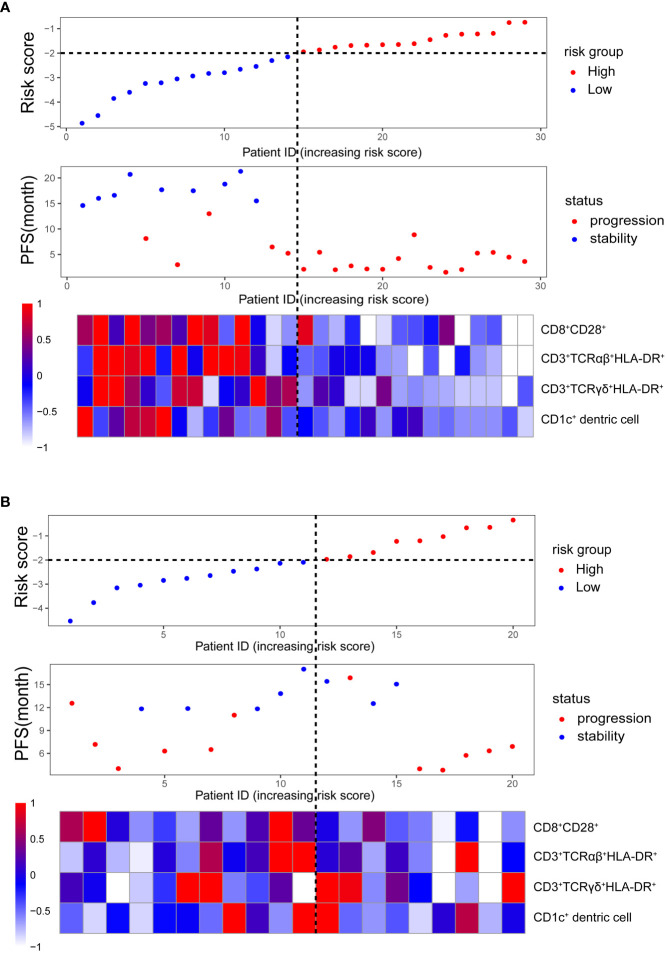
Clinical characteristics of malignant melanoma patients (in order from top to bottom): the risk score distribution in high- and low-risk groups; the progression-free survival status distribution increasing the risk score; the heatmap of the absolute counts of four-immune cell-subset profiles, representing the median value expressed by markers normalized to the range of 0 to 1. **(A)** Training cohort. **(B)** Validation cohort.

**Figure 3:**
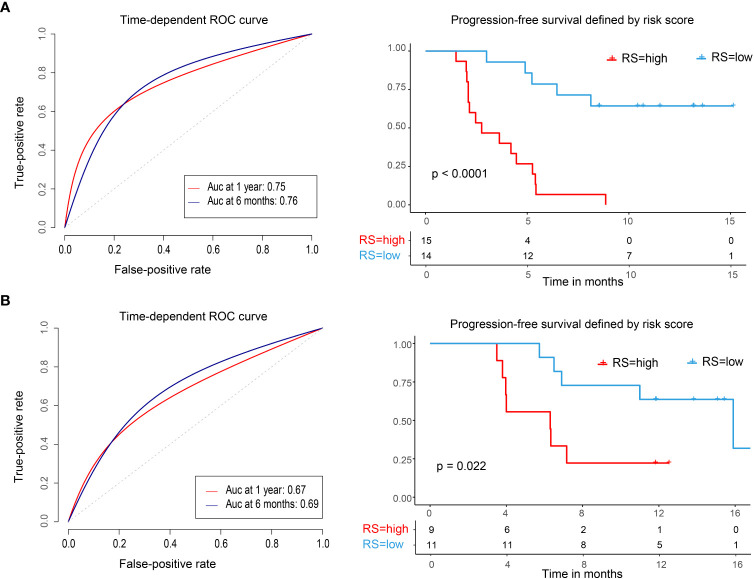
Time-dependent ROC curves, Kaplan–Meier survival curves in the training set and validation set. ROC, receiver operator characteristic; AUC, area under the curve. **(A)** Training cohort. **(B)** Validation cohort. We used AUCs at 0.5 and 1 years to assess prognostic accuracy and calculated P values using the log-rank test.

We used the median risk score as a cutoff to divide 29 patients into high-risk and low-risk groups. We used a risk plot to display the distribution of risk score, PFS, and disease status of all 29 patients (**Figure 2A**). It showed that with increased risk score, patients have worse PFS. We assessed prognostic accuracy of the four-immune cell-subset-based classifiers with a time-dependent ROC analysis at varying times (**Figure 3A**). The Kaplan–Meier plot represented patients in the high-risk group as having significantly shorter progression-free survival times than those in the low-risk group (*P* < 0.0001, **Figure 3A**). The mean progression-free survival of the high-risk group was 3.6 months (95% CI, 2.6–4.6 months) less than 12.3 months (95% CI, 9.9–14.7 months) of the low-risk group: they had better DCR (92.9% vs. 33.3%) and ORR (35.7% vs. 20.0%).

We did the same analyses with blood samples from the validation cohort (20 patients from the same center, **Figure 2B**). The four-immune cell-subset-based classifiers had excellent prognostic accuracy. The mean progression-free survival was 6.6 months (95% CI, 4.5–8.9 months) for high-risk groups and 13.2 months (95% CI, 10.5–15.9 months) for low-risk groups (*P* < 0.0001, **Figure 3B**).

### Independent prognostic factor evaluation and correlation with clinical characteristics

We used the median as a cutoff score of four subsets of immune cells in the LASSO Cox regression model. The appendix shows univariate analysis for disease-free survival of immune cell subsets for training and validation sets (**Table S3**).

**Figure 4:**
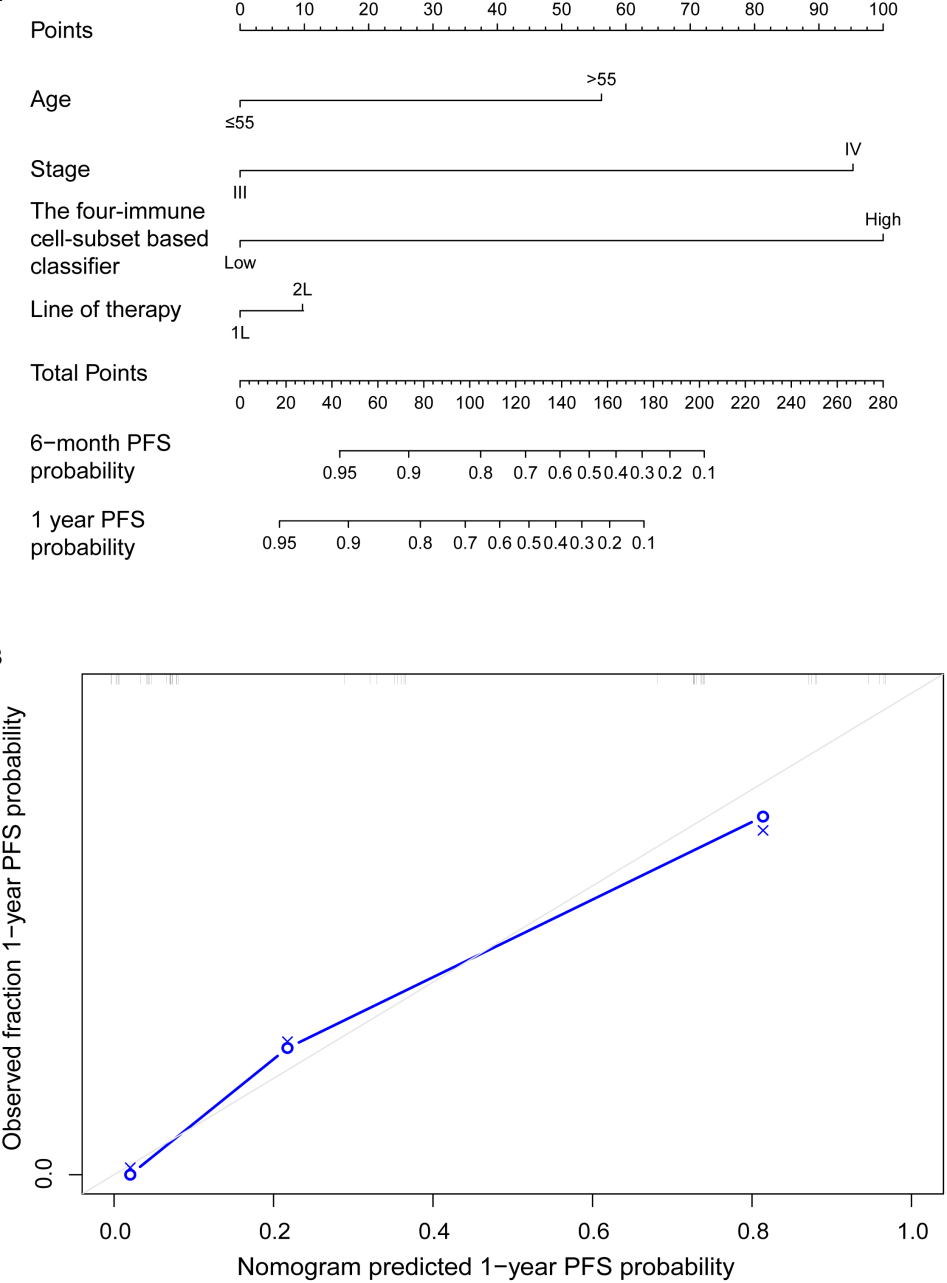
**(A)** Nomograms to predict risk of disease progression with anti-PD-1 inhibitor-based treatment in advanced malignant melanoma patients. **(B)** Plots depict the calibration of each model in terms of agreement between predicted and observed 1-year outcomes. Model performance was shown by the plot, relative to the 45° line, which represents perfect prediction.

To evaluate whether the immune risk score could be used as an independent prognostic factor, we assessed accuracy of the four-immune cell-subset-based classifiers with time-dependent ROC analyses. As indicated in **Figure S1**, the AUC of the immune risk score was largest (0.752) compared with the individual cell population. After multivariable adjustment by clinicopathologic variables, we used univariate and multivariate Cox regression to confirm that the four-immune cell-subset classifiers were powerful independent factors in the entire cohort of 49 cases (HR 5.897, 95% CI 2.489–13.971, *P* = 0.001, **Table 2**). We noted similar results in the validation set (HR 3.97, 1.124–13.586; *P* = 0.032; **Table S3**), as we created a prognostic nomogram to quantify the relationship between the immune risk score and the PFS. From this nomogram, we obtained total points and estimated the 6-month and 1-year survival rates of each patient (**Figure 4A**). The C-index was 0.796, whereas calibration curves (**Figure 4B**) clarified the accuracy of this nomogram.

**Table 2:** Univariate and multivariate cox hazards analysis for progress-free survival in 49 patients with melanoma.

Characteristics	Univariate analysis		Multivariate analysis	
	Hazard ratio (95% CI)	P value	Hazard ratio (95% CI)	P value
Gender Male vs. female	0.618 (0.284-1.345)	0.225		
Age ≤60 vs. >60	0.650 (0.317-1.334)	0.240	1.408 (0.664-2.986)	0.373
ECOG 0 vs. 1	1.034 (0.491-2.174)	0.931		
LDH >ULN^*^ vs. ≤ULN^*^	2.039 (0.703-5.915)	0.190	1.376 (0.453-4.181)	0.573
Histology				
Acral	ref	0.664		
CSD^**^	1.434 (0.651-3.158)	0.371		
Other	1.109 (0.418-2.940)	0.836		
TNM stage III vs. IV	13.755 (1.867-101.322)	0.01	11.181 (1.467-85.259)	0.02
Line of therapy ≥2 line vs. 1 line	1.966 (0.870-4.443)	0.104	1.214 (0.517-2.854)	0.656
Ulcer Ulceration vs. non-ulceration	0.836 (0.412-1.698)	0.621		
CD8^+^CD28^+^ T cell High vs. low	0.658 (0.292-1.481)	0.312		
CD3^+^TCRab^+^HLA-DR^+^ T cell High vs. low	0.793 (0.365-1.496)	0.4		
CD3^+^TCRrd^+^HLA-DR^+^ T cell High vs. low	0.553 (0.272-1.124)	0.101		
CD1c^+^ dendritic cell High vs. low	0.734 (0.362-1.490)	0.392		
Immune risk score†† High vs. low	7.036 (3.052-16.217)	0.001	5.897 (2.489-13.971)	0.001

^*^ULN, upper limit of normal. ^**^CSD, chronic sun damage. ^††^Immune risk score refers to the four-immune cell-subset based classifier, including three subpopulations of T cells (CD8^+^CD28^+^, CD3^+^TCRαβ^+^HLA-DR^+^, CD3^+^TCRγδ^+^HLA-DR^+^) and CD1c^+^ dendritic cells.

### Extended analysis of four immune cell-subset gene signatures

**Figure 5:**
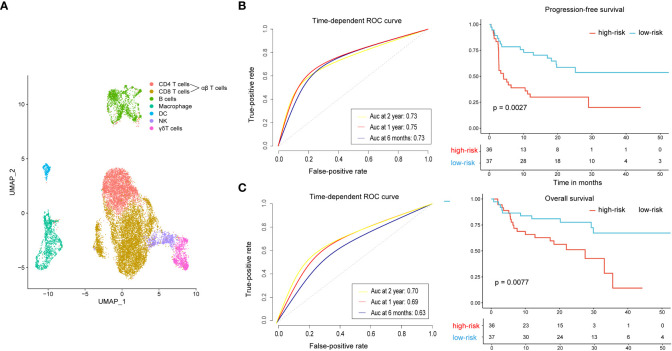
Extended analysis of the four-immune cell-subset gene signature on an independent dataset. **(A)** UMAP displays all immune cells collected in the GEO cohort. **(B)** Time-dependent ROC curves and Kaplan–Meier survival curves of progression-free survival. **(C)** Time-dependent ROC curves and Kaplan–Meier survival curves of overall survival. To assess prognostic accuracy, we used AUCs at 0.5, 1, and 2 years and calculated P values using the log-rank test. ROC, receiver operator characteristic; AUC, area under the curve.

We used the single-cell RNA sequencing dataset (GSE120575) to identify marker genes of CD8^+^ T cell, αβT cell, γδT cell, and DC clusters. The feature gene signature for the four features were clustered as described above. The final clustering was visualized as a UMAP (**Figure 5A**). To confirm the value of these four subsets of immune cells in the prognosis of malignant melanoma treated with PD-1 blockade, we applied it to the independent validation set of 73 advanced melanoma patients treated with anti-PD-1 monotherapy (n = 41) or combined anti-PD-1 and anti-CTLA-4 (n = 32) (25). We created distinct gene signatures for the CD8^+^ T cell, αβT cell, γδT cell, and DC clusters, each made up of 13, 24, 7, and 203 genes, respectively (**Table S4**), and we utilized them to create a composite score, where a high value indicated that the tumor specimen under analysis had a high cell abundance. We used the median composite score as a cutoff to divide 73 patients into high-risk and low-risk groups. Notably, patients in the high-risk group had significantly shorter progression-free survival than patients in the low-risk group (*P* = 0.0027, **Figure 5B**). The high-risk group’s mean progression-free survival was 14.5 months (95% CI, 8.6–20.4 months), shorter than the low-risk group’s, which had higher DCR (81.1% vs. 58.3%) and ORR (75.7% vs. 33.3%). Additionally, the overall survival of patients in the low-risk group was longer than that in the high-risk group, 40.6 months (95% CI, 33.8–47.3 months) vs. 22.9 months (95% CI, 17.4–28.5 months), *P* = 0.0077 (**Figure 5C**). We assessed the four-immune cell-subset gene signature-based classifier’s prognosis accuracy using time-dependent ROC analysis at various follow-up times (**Figures 5B, C**).

## Discussion

Immunotherapy enhances a patient’s immune system to fight disease and, as such, has revolutionized cancer treatment. Among the many immunotherapeutic strategies, immune checkpoint blockades show remarkable benefits for treatment of various cancer types, such as PD-1/PD-L1, which is widely used in the treatment of melanoma patients. However, the subsequent immune-related adverse events (irAE) or low response to immune checkpoint blockades precludes a large proportion of melanoma patients in benefiting from this therapy (26). It is urgent to find accurate and accessible biomarkers in a scoring system that can predict efficacy and prognosis of the clinical application of ICIs. Performing the biopsy of pathological tissue before and during therapy is the most direct means of immune monitoring, whereas it is a rather impractical approach for most patients and clinical settings. Cancer is a systemic disease that induces functional and compositional changes in the immune system. Systemic immunity is required for effective cancer immunotherapy (27, 28). Immunity is regulated by interactions of diverse cell lineages across tissues, from the tumor microenvironment (TME) to the peripheral blood. Therefore, the analysis of blood samples beyond the biopsy seems more feasible and may even reflect the immunological environment in the tumor.

A series of recent studies identified multiple immune cell subsets captured in peripheral blood instead of the TME that supports systemic immune responses in immunotherapy monitoring for different tumor types. For example, CD8^+^CD28^+^ T cells were reported as an independent predictive biomarker for non-invasive early screening in NSCLC occurrence and progression (29). When compared with healthy volunteers, a decrease in the CD3^+^TCRγδ^+^ lymphocyte subset was observed in multiple myeloma patients (30). CD3^+^HLA-DR^+^-activated T cells could determine the prognostics of lymphoma patients (31). A recent study found that peripheral T-cell and classically activated (M1) macrophage enrichment is associated with long-term clinical benefits of PD-1 mAb in NSCLC (32). The frequency of CD1c^+^ dendritic cells could predict the progression-free survival of renal cell cancer patients (11). Distinct immune signatures, such as CD8^+^PD-1^+^ T cells, CD8^+^ effector memory (CD8^+^CD45RA^−^CD45RO^+^CCR7^−^) T cells, activated CD4^+^ T cells (CD4^+^CD38^+^HLA-DR^+^), and NK cells (CD16^+^CD56^+^CD38^+^HLA-DR^+^) showed the treatment response and immune-related adverse events in melanoma patients in ICI therapy (33). However, the disadvantage of previous studies is the limited immune cell subsets or the inappropriate use of statistical methods to analyze data. In our study, immune cell subsets of peripheral blood were included for detection, with up to 66 subpopulations, i.e., cells of innate immunity and adaptive immunity. The LASSO Cox regression model in this study can integrate multiple immune cell subsets into one tool, providing significantly greater prognostic accuracy than a single immune cell subset alone. Our results showed that four-immune-cell subsets which involved innate immunity (CD1c^+^ dendritic cells and CD3^+^TCRγδ^+^HLA-DR^+^ T cells) and adaptive immunity (CD8^+^CD28^+^ and CD3^+^TCRαβ^+^HLA-DR^+^ T cells) could successfully categorize patients into high- and low-risk groups with differences in 1 year of progression-free survival. This immune risk score can predict the progression-free survival of those in stage III/IV melanoma more than clinicopathologic risk factors and single immune cell subpopulations. It is a prognostic model that complements clinicopathologic features.

Immune response is a dynamic process whose nature and intensity vary over time. It begins with the antigen-independent responses of innate immunity and becomes more powerful as the antigen-specific adaptive immune response matures. The innate immune response sets the scene for induction of an adaptive immune response, orchestrated by signals that emanate from innate sensor cells and are coordinated with innate effector cells to yield pathogen clearance (34). Akin to PD-1 immunotherapy, even though this therapy is applied from T-cell basic science to clinical practice (belonging to the scope of adaptive immunity), effective immunotherapy cannot be achieved without the participation of innate immune cells, such as dendritic cells and NK cells (27, 28). Dendritic cells and antigen-presenting cells (APC) are the most important innate immune factors associated with initiating T-cell responses in cancer, as they interact to orchestrate an overall immune response. Growing evidence has verified that systemic dendritic cell dysfunction is a cause of blunted CD8^+^ T-cell proliferation and differentiation in cancer, as promoting dendritic cell activation can rescue CD8^+^ T-cell activity. Reduction of dendritic cell apoptosis and repair of dendritic cell maturation drove superior control of tumor growth (35, 36). In human peripheral blood, there are two major subsets of DCs, myeloid DCs (mDCs), and plasmacytoid DCs (pDCs). The mDCs (lineage^-^HLA-DR^+^CD11c^+^CD123^low^) play a major role in antigen capture, presenting T cells. The mDCs are further subdivided into three subsets, the CD1c^+^ (BDCA-1^+^) mDCs, the CD141^+^ (BDCA-3^+^) mDCs, and the CD16^+^ mDCs (37–39). The CD1c^+^ mDCs are primarily involved in the presentation of lipid and glycolipid antigens to T cells while expressing low levels of PD-L1 antigen (40). Thus, it is reasonable that the CD1c^+^ mDCs can predict the effect of PD-1 treatment in our study. Prior research showed that low levels of dendritic cells at the beginning of therapy are linked to primary resistance to checkpoint blockade monotherapy (41). The accurate quantification of dendritic cells in our investigation could offer some insight into their interaction with PD-1 immunotherapy. CD8+CD28+ T cells are another immune cell subset involved in the prognostic model of our research. CD28 is the co-stimulatory receptor expressed on the surface of T cells. Early after activation, generally in the lymphoid tissue, T cells are activated when their TCRs bind to the cognate antigen presented by APCs in conjunction with CD28 binding to B7-1/B7-2 (42). This binding of CD28 to B7-1/B7-2 determines the activation of T cells. Previous studies revealed that rescuing CD8+ T-cell cytotoxicity by a PD-1 blockade depended on the expression of CD28 as PD-1-mediated immunomodulation was lost in the context of CD28 conditional knockout mice. Moreover, reinvigorated T cells in patient peripheral blood with lung cancer following PD-1 blockade were shown to predominantly express CD28 (43). This demonstrated that the CD28/B7 co-stimulatory pathway was essential for effective PD-1 therapy. We found that CD1c^+^ mDCs, CD8^+^CD28^+^ T cells, and PD-1 inhibitors interact with each other to achieve optimal therapeutic effect.

The risk score of our research includes two subsets of activated T lymphocytes, CD3^+^TCRαβ^+^HLA-DR^+^ T cells and CD3^+^TCRγδ^+^HLA-DR^+^ T cells. Expression of HLA-DR in T cells indicates late activation (44, 45) but was found to be linked to certain clinical morphologic features. The expressions of CD4^+^HLA-DR^+^ markers were higher for pT3 and pT4 tumors, compared with pT2 laryngeal carcinomas. In addition, more aggressive and deeply infiltrating laryngeal carcinomas were characterized by significantly higher values of the average expression of the HLA-DR marker on CD4^+^ T cells (46). Also, αβT and γδTCR cells can be distinguished by the expression of either αβTCR or γδTCR, respectively. Despite some similarities in the structure of TCRαβ and TCRγδ and the shared subunits of the CD3 complex, the two receptors differ in important aspects, such as the glycosylation pattern, the assembly geometry of the complex, the plasma membrane organization, and accessibility of signaling motifs in the CD3 intracellular tails (47). These differences could impact the activation mechanism of the two TCRs. While the architecture of αβTCRs is specialized in the recognition of MHC, γδTCRs bind to many different ligands. There are two populations of αβT cells, CD4^+^ T cells and CD8^+^ T cells; the former recognizes peptides presented in MHC class II, whereas the latter recognizes peptides in MHC class I. In contrast, the majority of γδTCRs lacking CD4 and CD8 expression do not recognize MHC molecules but corresponding ligands with their own expressed receptors. In addition, γδT cells might enable a strong proliferative response to TCR stimulation in the absence of CD28 co-stimulation, which might be linked to contributing functions to initiate an immune response (48). Moreover, αβT cells are involved in adaptive immune responses, whereas γδT cells are non-conventional lymphocytes, with several properties of innate immune cells while serving as the bridge to connect the innate and adaptive immune systems (49). In human peripheral blood, αβT cells typically represent more than 90% of all T lymphocytes, whereas γδT cells only represent 1%–10%. However, γδT cells are widely localized in non-lymphoid tissues and constitute immune cells on epithelial surfaces, where they participate in the maintenance of epithelial barriers (50). Although the theoretical basis of PD-1 therapy is derived from the activation mechanism of αβT cells, PD-1 inhibitors also affect γδT cells. Myeloid cells induced γδT cell exhaustion through PD-L1 expression, and the PD-1/PD-L1 axis downregulated IFN-γ production and antibody-dependent cellular cytotoxicity (ADCC) of γδT cells (51–53).

PD-1/PD-L1 blockade therapy is the bench-to-bedside approach connecting basic science with clinical practice. Our results show that the risk scores of innate immune and adaptive cell populations predict the prognosis of melanoma patients treated with anti-PD-1. It shows that although anti-PD-1 therapy is based on activation mechanisms of T cells in adaptive immunity to achieve a therapeutic effect, innate immune cells are also necessary.

In this study, we developed a novel prognostic tool based on four immune cell subsets existing in peripheral blood to improve the prediction of disease progression of stage III/IV melanoma patients in South China treated with anti-PD-1 antibody. As far as we know, our study is the first based on absolute counts of immune cells to construct a multi-immune cell subset prognostic model index to predict the progression-free survival of advanced malignant melanoma patients treated with anti-PD-1 inhibitors. Collectively, our results identify patients who are more likely to respond to PD-1 antibodies and remind potential non-responders to pursue effective treatment options in a timely way. The majority of patients in this study were from South China, most of whom were of acral or mucosal type after failure of first-line treatment. More than half of these patients received PD-1 inhibitors combined with chemotherapy, which is the off-label regimen. Therefore, the application of this immune risk score in Caucasian populations predominantly with a cutaneous type needs to be further verified. Furthermore, there are no sufficient public data to validate the four-immune cell-subset-based classifiers, rendering it vulnerable to biases inherent in this study design. We used public databases of single-cell transcriptome data of human melanoma samples to construct a composite score based on the four immune-cell subsets. Notably, it had shown good performance in predicting PFS. This result supports the value of the four immune cell subsets in identifying potential non-responders of anti-PD-1 antibody timely. However, its interpretive power for models constructed based on flow cytometry is limited as they are different omics data. Additional prospective research in multicenter clinical trials must confirm our findings.

## Data availability statement

The raw data supporting the conclusions of this article will be made available by the authors, without undue reservation.

## Ethics statement

The studies involving human participants were reviewed and approved by Ethics Committee of Sun Yat-sen University Cancer Center. The patients/participants provided their written informed consent to participate in this study.

## Author contributions

YD and DL did the study design and concepts. JL, XW, QD, and LC did the data acquisition. PC and HJ did the data processing and wrote the draft of the paper. XZ, PC, YD, and HJ checked and revised the first draft of the paper. HJ did the statistical analysis and manuscript editing. All authors contributed to the article and approved the submitted manuscript.
