# Supplementary material for: An immune risk score predicts progression-free survival of melanoma patients in South China receiving anti-PD-1 inhibitor therapy—a retrospective cohort study examining 66 circulating immune cell subsets
Source: Front Immunol. 2022 Dec 7;13:1012673. doi: 10.3389/fimmu.2022.1012673 (PMC9768215; doi:10.3389/fimmu.2022.1012673)
Supplement: Supplementary file 1 [file DataSheet_1.docx]

Supplementary Material

## Supplementary Figures


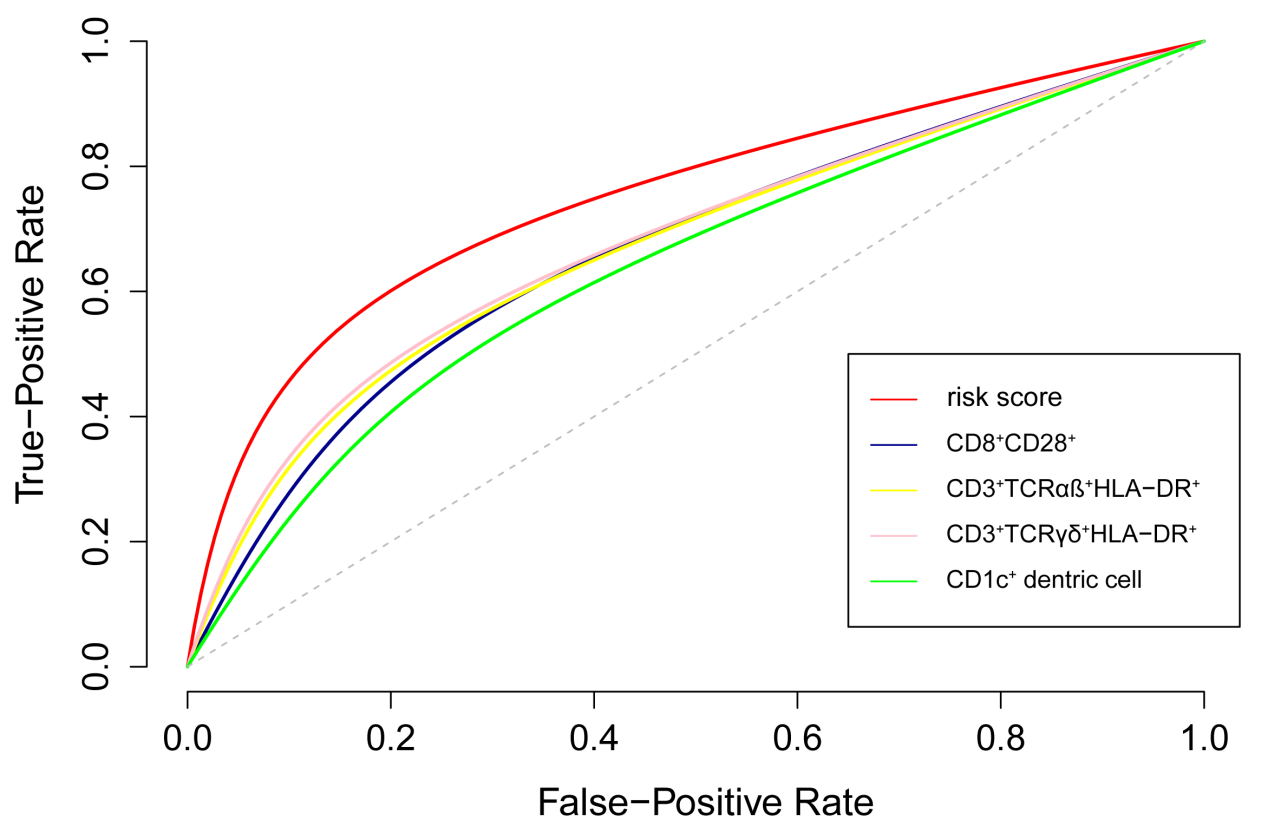


**Supplementary Figure 1.** Time-dependent ROC curves compared the prognostic accuracy of the four-immune cell- subset based classifier with single immune cell subpopulation in training set.

## Supplementary Tables

**Supplementary Table S1** Detection panels of the immune cell subsets in peripheral blood by flow cytometry

| Panels | 405nm | | 488nm | | | | | 633nm | | |
| --- | --- | --- | --- | --- | --- | --- | --- | --- | --- | --- |
|  | Pacific Blue | Krome Orange | FITC | PE | ECD | PC5.5 | PC7 | APC/  A647 | A700/  APC-A700 | APC-A750 |
| Basic tube | - | CD45 | CD16 | CD56 | CD19 | - | CD14 | CD4 | CD8 | CD3 |
| T cell subsets tube | CD57 | CD45 | CD45RA | CCR7 | CD28 | PD1 | CD27 | CD4 | CD8 | CD3 |
| TCR subsets tube | TCRVd2 | CD45 | TCRγδ | TCRαβ | HLA-DR | - | TCRVd1 | CD4 | CD8 | CD3 |
| Treg tube | - | CD45 | CD45RA | CD25 | - | CD39 | CD4 | CD127 | - | CD3 |
| B cell tube | IgM | CD45 | IgD | CD21 | C19 | - | CD27 | CD24 | - | CD38 |
| Dentric cell tube | HLA-DR | CD45 | CD16 | Lineage§ | - | CD1c | CD11c | Clec9A | CD123 | - |

§: CD3/CD19/CD20/CD14/CD56

**Supplementary Table S2** Details of 66 circulating immune cell subsets detected in this study

| **lymphocyte basic subsets and monocyte subsets** | |
| --- | --- |
| **Common name of the subsets** | **The gated strategy** |
| Leucocytes | CD45^+^SS^high^ |
| Classical Monocytes | CD14^high^CD16^-^ |
| Intermediate Monocytes | CD14^high^CD16^+^ |
| Non-Classical Monocytes | CD14^-^CD16^high^ |
| Lymphocytes | CD45^+^SS^low^FS^low^ |
| B Cells | CD3^-^CD19^+^ |
| Natural killer (NK) cells | CD3^-^CD56^+^ |
| NKT cells | CD3^+^CD56^+^ |
| CD56^dim^ NK cells | CD56^dim^CD16^+^ |
| CD56^bri^ NK cells | CD56^bri^CD16^+/-^ |
| T cell subsets | |
| **Common name of the subsets** | **The gated strategy** |
| Total T lymphocytes | CD3^+^ |
| CD4^+^ T cell | CD3^+^CD4^+^ |
| CD8^+^ T cell | CD3^+^CD8^+^ |
| DP T | CD4^+^CD8^+^ |
| DN T | CD4^-^CD8^-^ |
| CD4^+^ Naive T cell | CD3^+^CD4^+^CD45RA^+^CCR7^+^ |
| CD4^+^ Central Memory T cell | CD3^+^CD4^+^CD45RA^-^CCR7^+^ |
| CD4^+^ Effector Memory T cell | CD3^+^CD4^+^CD45RA^-^CCR7^-^ |
| CD4^+^ Terminally differentiated effector memory T cell | CD3^+^CD4^+^CD45RA^+^CCR7^-^ |
| CD8^+^ Naive T cell | CD3^+^CD8^+^CD45RA^+^CCR7^+^ |
| CD8^+^ Central Memory T cell | CD3^+^CD8^+^CD45RA^-^CCR7^+^ |
| CD8^+^ Effector memory T cell | CD3^+^CD8^+^CD45RA^-^CCR7^-^ |
| CD8^+^ Terminally differentiated effector memory T cell | CD3^+^CD8^+^CD45RA^+^CCR7^-^ |
| CD4^+^CD27^+^ T cells | CD3^+^CD4^+^CD27^+^ |
| CD4^+^CD28^+^ T cells | CD3^+^CD4^+^CD28^+^ |
| CD4^+^PD-1^+^ T cell | CD3^+^CD4^+^PD-1^+^ |
| CD4^+^CD57^+^ T cells | CD3^+^CD4^+^CD57^+^ |
| CD8^+^CD27^+^ T cells | CD3^+^CD8^+^CD27^+^ |
| CD8^+^CD28^+^ T cell | CD3^+^CD8^+^CD28^+^ |
| CD8^+^PD-1^+^ T cell | CD3^+^CD8^+^PD-1^+^ |
| CD8^+^CD57^+^ T cells | CD3^+^CD8^+^CD57^+^ |
| TCR subsets | |
| **Common name of the subsets** | **The gated strategy** |
| αβT cells | CD3^+^TCRαβ^+^ |
| γδT cells | CD3^+^TCRγδ^+^ |
| Vδ1 T cells | CD3^+^TCRγδ^+^TCRVδ1^+^ |
| Vδ2 T cells | CD3^+^TCRγδ^+^TCRVδ2^+^ |
| Activated CD4^+^ T cells | CD3^+^CD4^+^HLA-DR^+^ |
| Activated CD8^+^ T cells | CD3^+^CD8^+^HLA-DR^+^ |
| Activated αβT cells | CD3^+^TCRαβ^+^HLA-DR^+^ |
| Activated γδT cells | CD3^+^TCRγδ^+^HLA-DR^+^ |
| Activated Vδ1 cells | CD3^+^TCRγδ^+^TCRVδ1^+^HLA-DR^+^ |
| Activated Vδ2 cells | CD3^+^TCRγδ^+^TCRVδ2^+^HLA-DR^+^ |
| CD4^+^ αβT cells | CD3^+^TCRαβ^+^CD4^+^ |
| CD8^+^ αβT cells | CD3^+^TCRαβ^+^CD8^+^ |
| DP αβT cells | CD3^+^TCRαβ^+^CD4^+^CD8^+^ |
| DN αβT cells | CD3^+^TCRαβ^+^CD4^-^CD8^-^ |
| CD8^+^ γδT cells | CD3^+^TCRγδ^+^CD8^+^ |
| DN γδT cells | CD3^+^TCRγδ^+^CD4^-^CD8^-^ |
| Treg subsets | |
| **Common name of the subsets** | **The gated strategy** |
| CD25^+^ activated CD4^+^ T cells | CD4^+^CD25^+^ |
| Treg cells | CD4^+^CD25^+^CD127^dim^ |
| Naïve Treg cells | CD4^+^CD25^+^CD127^dim^CD45RA^+^ |
| Memory Treg cells | CD4^+^CD25^+^CD127^dim^CD45RA^-^ |
| CD39^+^ Treg cells | CD4^+^CD25^+^CD127^dim^CD39^+^ |
| CD39^-^ Treg cells | CD4^+^CD25^+^CD127^dim^CD39^-^ |
| B cell subsets | |
| **Common name of the subsets** | **The gated strategy** |
| Transitional B cells | CD19^+^IgM^+^CD27^-^CD24^+^CD38^high^ |
| Naive B cells | CD19^+^CD27^-^IgD^+^ |
| Marginal zone B cells | CD19^+^CD27^+^IgD^+^ |
| Non switched memory B cells | CD19^+^IgM^+^CD27^+^CD38^dim^ |
| Class switched memory B cells | CD19^+^IgM^-^IgD^-^CD27^+^CD38^dim^ |
| Plasmablasts | CD19^+^IgM^-^IgD^-^CD27^high^CD38^high^ |
| Memory B cells | CD19^+^CD27^+^CD38^dim^ |
| CD21^-^ B cells | CD19^+^CD21^low^CD38^low^ |
| Dendritic cell subsets | |
| **Common name of the subsets** | **The gated strategy** |
| Dendritic cells | CD45^+^Lin^-^HLA-DR^+^ |
| Plasmacytoid dendritic cells | CD45^+^Lin^-^HLA-DR^+^CD123^+^ |
| Myeloid dendritic cells | CD45^+^Lin^-^HLA-DR^+^CD11c^+^ |
| CD11c^+^ myeloid dendritic cells | CD45^+^Lin^-^HLA-DR^+^CD11c^+^CD1c^+^ |
| CD16^+^ myeloid Dendritic cells | CD45^+^Lin^-^HLA-DR^+^CD11c^+^CD16^+^ |

**Supplementary Table S3** Univariate cox hazards analysis for progress-free survival of the training set and validation set

| Characteristics |  | Training set | |  | Validation set | |
| --- | --- | --- | --- | --- | --- | --- |
|  |  | Hazard ratio (95% CI) | P value |  | Hazard ratio (95% CI) | P value |
| Gender  Male vs Female |  | 0.537 (0.156-1.85) | 0.325 |  | 0.875 (0.281-2.724) | 0.818 |
| Age  ≤60 vs ＞60 |  | 1.213 (0.458-3.21) | 0.698 |  | 0.535 (0.156-1.835) | 0.320 |
| ECOG  0 vs 1 |  | 0.838 (0.329-2.135) | 0.712 |  | 1.300 (0.379-4.459) | 0.676 |
| LDH  ≤ ULN^*^ vs ＞ ULN ^*^ |  | 1.325 (0.436-4.028) | 0.620 |  | 3.011 (0.635-14.270) | 0.165 |
| Histology |  |  |  |  |  |  |
| Acral |  | Ref | 0.416 |  | Ref | 0.631 |
| CSD^**^ |  | 0.519 (0.179-1.506) | 0.227 |  | 0.422 (0.052-3.403) | 0.418 |
| Other |  | 1.175 (0.321-4.296) | 0.808 |  | 0.644 (0.170-2.449) | 0.519 |
| TNM stage  Ⅲ vs Ⅳ |  | 8.879 (1.175-67.101) | 0.034 |  | 37.882 (0.195-7368.7) | 0.177 |
| Line of therapy  1 Line vs ≥2 Line |  | 1.585 (0.57-4.411) | 0.378 |  | 2.757 (0.702-10.834) | 0.146 |
| Ulcer  Ulceration vs Non-ulceration |  | 0.763 (0.309-1.884) | 0.558 |  | 1.159 (0.347-3.870) | 0.810 |
| CD8^+^CD28^+^ T cell  High vs Low |  | 0.307 (0.114-0.825) | 0.019 |  | 0.417 (0.088-1.975) | 0.270 |
| CD3^+^TCRab^+^HLA-DR^+^ T cell  High vs Low |  | 0.443 (0.172-1.138) | 0.091 |  | 0.545 (0.163-1.819) | 0.323 |
| CD3^+^TCRrd^+^HLA-DR^+^ T cell  High vs Low |  | 0.345 (0.134-0.888) | 0.027 |  | 0.771 (0.231-2.574) | 0.672 |
| CD1c^+^ dendritic cell  High vs Low |  | 0.395 (0.154-1.017) | 0.054 |  | 0.610 (0.193-1.933) | 0.401 |
| Immune risk score††  High vs Low |  | 10.362 (3.234-33.204) | 0.001 |  | 3.970 (1.124-13.586) | 0.032 |

* ULN, upper limit of normal, **CSD, chronic sun damage, ††Immune risk score referred to the four-immune cell-subset based classifier, including three subpopulations of T cell (CD8^+^CD28^+^, CD3^+^TCRαβ^+^HLA-DR^+^, CD3^+^TCRγδ^+^HLA-DR^+^) and CD1c^+^ dendritic cell.

**Supplementary Table S4** Details of four-immune cell-subset gene signature

| Signatue | type | Marker genes | Publication |
| --- | --- | --- | --- |
| Dendritic_cells | mRNA | LILRA4, DERL, TCL1A, POLB, GSN, SRC, NCF1C, RP11-73G16.2, PLEKHD1, SERPINF1, CSF2RB, ZB1, CHAF1A, RP1-257A7.5, PTPRE, HVCN1, MAP2K6, IRF2BP2, PLD4, TNFRSF21, P2RY6, AC010441.1, SH2B3, ST3GAL4, HLA-DMB, RNU2-2P, RP11-1100L3.7, IGJ, APP, FAM129C, TNFSF13, THBD, RRBP1, IGHM, LDLRAD4, TXN, IL3RA, NRP1, P2RY14, IRF7, SMIM5, ALG2, SYK, TXNDC5, CD2AP, RP11-38J22.6, CLIC3, MYBL2, IGKC, TRAF4, C12orf45, CD83, UBE2E2, SCARB2, PTPRS, C1orf186, MS4A6A, UGCG, MGST2, GZMB, NR4A3, PHEX, ARID3A, SPIB, TYROBP, CD36, GNA15, TLR7, SIRPB1, GPX1, GNG7, ERN1, TSPAN13, CD68, CORO1C, CYBB, SEMA7A, GRASP, AMIGO3, ETV3, BAIAP2, SMPD3, FCER1G, PLAC8, ALDH2, CLN8, CD4, VIMP, SPG20, SEPHS1, EGLN3, PTCRA, GAS6, SLC12A3, GAB1, DPYSL2, PPM1J, PHACTR1, DSTN, PTGDS, SOX4, WDFY4, PACSIN1, PLEK, FCGRT, AREG, BID, LILRB4, PFKFB2, SUSD1, RASD1, NREP, MEF2C, DCK, TP53I11, NAPSB, LAMP5, GRN, KRT5, ZDHHC17, PALD1, TUBB6, AMICA1, TCF4, SLC15A4, ITM2C, SULF2, AC023590.1, FGD2, RASSF2, SEL1L3, MPEG1, TPM2, RNF130, IDH3A, CIITA, CAPG, RAB11FIP1, AFF3, CST3, EPHB1, THEMIS2, NOTCH4, C12orf75, CBFA2T3, NCF1B, TNFSF13B, DNASE1L3, RP11-117D22.2, BLNK, SPINT2, FCHSD2, NEK8, C12orf44, EIF2AK4, TGFBI, CCDC50, RP11-71G12.1, CCDC88A, HIGD1A, PLVAP, NCF1, FADS1, AC096579.7, LGMN, SCAMP5, CSF2RA, ST6GALNAC4, RUNX2, TSPAN3, SCN9A, CLEC4C, CYB561A3, NPC2, GPR183, P2RX1, FAM65A, C3orf58, SMIM14, RNASE6, TLR9, MAP1A, IRF4, KIAA0226L, ENPP2, TEX2, LAIR1, BCL11A, IRF8, GAPT, PLXNB2, CTNS, SLC41A2, HYOU1, VEGFB, ZFAT, LINC00996, PPP1R14B, FAM213A, FLNB, SERPING1, ATF5, HIVEP1 | PMID: 30388456 |
| αβT_cells | mRNA | CD8A, TRAC, CD8B, CD3D, CD3G, CD2, CTLA4, IL32, ITM2A, ICOS, CD3E, PDCD1, CXCL13, TIGIT, TRBC2, ITK, INPP4B, DUSP4, CD5, CD6, LCK, CD28, SIRPG, FYN |  |
| γδT_cells | mRNA | TRDC, TRDV1, TRDV2, TRGC2, TRGC1, AE000661.37, KLRG1 |  |
| CD8+ T_cells | mRNA | CD8A, CD8B, NKG7, CCL5, KLRK1, GZMK, GZMA, CCL4, GZMH, PRF1, CST7, CTSW, CCL4L1 |  |

**Construction of a four-immune cell-subset based classifier with LASSO Cox regression**

A prevalent approach for regression with high-dimensional predictors would be LASSO. The penalty parameter, also known as the tuning parameter, governs the degree of shrinkage: the higher the value, the fewer predictors are chosen. LASSO has been expanded and widely used with the Cox proportional hazard regression model for high-dimensional data survival analysis. LASSO can be used to prevent overfitting by selecting biomarkers with high prognostic value and a low correlation among themselves with high-dimensional microarray data. To accomplish shrinkage and variable selection simultaneously, we used a Cox regression model with a LASSO penalty. Ten-time cross validations were used to establish which value was adequate. We decide based on minimum (Partial Likelihood Deviance) criteria, indicating that the parameter lambda should be set to its ideal value when partial likelihood deviance is at its lowest. We plotted the partial likelihood deviance vs log (λ), where the tuning parameter is located. A valueλ=0.22 with log (λ)=-1.51 was chosen by cross-validation via the minimum partial likelihood criteria (Figure 1B). The best tuning parameter produced four non-zero coefficients. Four-immune cell-subsets —CD8^+^CD28^+^, CD3^+^TCRαβ^+^HLA-DR^+^, CD3^+^TCRγδ^+^HLA-DR^+^—with coefficients 1.798, 4.06, 26.46, 169, respectively were selected for LASSO Cox regression (Figure 1A).
